# Postoperative cognitive dysfunction after robot-assisted radical cystectomy (RARC) with cerebral oxygen monitoring an observational prospective cohort pilot study

**DOI:** 10.1186/s12871-019-0877-5

**Published:** 2019-11-06

**Authors:** Yue Li, Dan Huang, Diansan Su, Jie Chen, Liqun Yang

**Affiliations:** Department of Anesthesiology, Renji Hospital, School of Medicine, Shanghai Jiaotong University, 160 Pujian Road, Shanghai, 200127 China

**Keywords:** Postoperative cognitive dysfunction, Robot-assisted radical cystectomy, Cerebral oxygen saturation

## Abstract

**Background:**

The incidence and risk factors of postoperative cognitive dysfunction (POCD) during robot-assisted radical cystectomy (RARC) in extreme Trendelenburg positioning and pneumoperitoneum are still controversial. The aim of this prospective observational study was to find the incidence rate as well as possible risk factors of POCD in RARC with cerebral oxygen monitoring.

**Methods:**

Patients who underwent RARC and open abdominal surgery in horizontal positioning were included. Preoperative and postoperative arterial blood gas (ABG), S-100β, C-reactive protein (CRP), and cognitive dysfunction scales were tested. Also, we used Z score to analyze and comprehensively evaluate POCD. Measurements of heart rate (HR), mean arterial pressure (MAP), central venous pressure (CVP), end-tidal CO_2_ (etCO_2_), and cerebral oxygen were immediately obtained after different time points during the surgery.

**Results:**

Here, 24 and 23 patients were included in the RARC group and in the control group, respectively. The incidence of POCD didn’t have significant difference in RARC group (45.8%), in contrast to the control group (26.1%). The laboratory tests of s100β and CRP between two groups didn’t contain significant difference as well. As duration of Trendelenburg and pneumoperitoneum prolonged, the cerebral oxygen saturation in the RARC group increased, which didn’t cause excessive perfusion nevertheless (rSO_2_<75%). We compared laboratory tests, age, education status, blood loss, and fluid input between POCD and non-POCD patients. A significant difference was found in the serum concentrations of CRP (72.59 ± 42.09 vs. 48.50 ± 26.53, *P* = 0.025) and age (69.20 ± 7.033 vs. 65.34 ± 5.228, *P* = 0.041).

**Conclusion:**

RARC in extreme Trendelenburg positioning and pneumoperitoneum did not significantly increase the incidence of POCD and didn’t cause excessive perfusion. The inflammation marker CRP and age might be independent risk factors of POCD.

**Trial registration:**

Clinicaltrials.gov with registration number NCT03372135. Registered 1 November 2017 (retrospectively registered).

## Background

Bladder cancer is one of the most common malignancies throughout the world [1] due to its high morbidity and mortality [2, 3]. There is an increasing intention in the use of robot-assisted radical cystectomy (RARC) because of improved short-term outcome in addition to functional results [4, 5]. One of its potential disadvantages is the necessity of extreme Trendelenburg position and CO_2_ pneumoperitoneum, which lead to complex pathophysiological changes [6, 7].

Pneumoperitoneum can cause an increase in intracranial pressure (ICP), which is partly related to an decreased drainage of the lumbar venous plexus [8, 9]. It can be explained by the modified Monroe-Kelly doctrine [9] that a change in one or more compartments of the intracranial space leads to a compensatory change in the remaining components. If changes would be occurred too fast to compensate, the ICP rises. Accordingly, pneumoperitoneum may bring about an increase in the ICP since the lumbar venous plexus reflux decreases.

Increased intra-abdominal pressure (IAP) can exert mechanical compression on vessels. A small decrease in the vessel diameters may lead to a remarkable decrease of the blood flow. Increased IAP may also stimulate the release of vasopressin, which can induce vasoconstriction. In addition, the brain is extremely sensitive to the change of pCO_2_. Prolonged time of pneumoperitoneum caused by carbon dioxide retention or even hypercapnia, may also lead to increase of intracranial pressure secondary to cerebral vascular expansion. The ICP elevation along with pneumoperitoneum further increases in the extreme Trendelenburg positioning, which may rise the risk of brain edema, blood-brain barrier damage, as well as potential brain injury caused by the decrease of cerebral perfusion and insufficient supply of oxygen.

Therefore, surgery in a steep Trendelenburg position and pneumoperitoneum might also affect cerebral integrity due to increased ICP and potential cerebral edema formation [10, 11]. These effects may possibly result in abnormal brain perfusion as well as anomalous cerebral oxygen supply [12, 13]. Additionally, the prolonged duration of operation (at least 3 h for extreme Trendelenburg positioning and pneumoperitoneum) may exacerbate the effects on elderly patients with relatively weak vascular walls [6, 10], which may result in POCD. Near-infrared spectroscopy (NIRS) can assist us to observe the position of cerebral perfusion. It is a relatively new technology for monitoring non-invasive regional cerebral oxygen saturation (rSO_2_). NIRS can penetrate to the brain with a depth of 1~2 cm [14] in a certain range (650–1100 nm). In the brain, the arteries and veins are staggered (veins 75%, arteries 20%, and capillaries account for 5%), indicating that the value of local cerebral oxygen saturation mainly represents the oxygen content in the venous blood, reflecting the cerebral oxygen transportation and metabolism. Recent studies have shown that decreased cerebral oxygen value (more than 25% of the base value) can increase the incidence rate of multiple organ dysfunction syndrome (MODS) and prolong ICU as well as average length of stay in hospital. The present study investigated the rSO_2_ value of the brain to observe the cerebral perfusion and oxygen metabolism during specific procedures.

POCD is a short-run decline in cognitive function after surgery, which may last from a few days to a few weeks. It is a common and influential outcome of surgical procedures in the aged. The mechanisms and pathophysiology for POCD have been poorly perceived. One possible biological mechanism for an impact on brain protein deposition may exist. However, the current knowledge is very limited. POCD is supposed to be associated with several factors such as age, trauma, inflammation, surgical stress, position, fluid, mean blood pressure, artificial pneumoperitoneum, and so on [15, 16]. With the advent of global aging and the development of robotic surgery, more elderly people will receive such surgical treatments, and the medical and social problems generated by POCD will become markedly serious.

Thus, our hypothesis was that the incidence of POCD in patients who underwent RARC would be higher than those who underwent open abdominal surgery in horizontal position. Accordingly, our primary aim was to observe the combined effect of Trendelenburg position and CO_2_ pneumoperitoneum on POCD during RARC with monitoring the cerebral oxygen. Our secondary aim was to determine probable risk factors of POCD.

## Methods

### Study design

This was an observational prospective cohort pilot study, which was approved by our Institutional Review Board and was registered by the Clinical Research Information Service (Clinical Trial.gov ID: NCT03372135).

### Subjects

The study was conducted between December 2017 and May 2018. Patients (with the age of more than 55 years old, ASA I-III) who underwent RARC and open abdominal surgery in horizontal position were included. All recipients were free of intracranial surgery and intracranial pathology (e.g., cerebral infarction). We also excluded patients who suffered from alcoholism or took psychotropic medication. Illiteracy, visual and hearing impairment and those who affected by low preoperative mini-mental state examination (MMSE) scores (less than 24) were eliminated as well.

### Preoperative and postoperative tests

Regarding preoperative cognitive dysfunction scales, C-reactive protein (CRP) and S-100β were tested. Laboratory variables of S-100β (brain injury biomarker) and CRP (an acute-phase protein in response to inflammation) were measured 48 h after surgery as well. Cognitive dysfunction scales were used to evaluate patients in the period of 1 week and 3 months [17] after surgery to compare with preoperative results. The test battery [18] included MMSE, a test of examination of functions including registration, attention and calculation, recall, language, ability to follow simple commands, and orientation; Brief Visuospatial Memory Test–Revised (BVMT-R), an assessment of non-verbal learning and memory; Symbol Digit Modalities Test, a test of psychomotor speed and attentional control; Trail Making Test (TMT), to assess visual scanning, psychomotor speed, attention, and executive function; Digit Span Test (DST), measuring attention and short-term verbal memory; and Stroop Color and Word Test (SCWT), a test for word-naming and resistance to interference. These tests were administered by experienced research personnel, who trained and supervised by a senior neuropsychologist.

### Position and insufflation during RARC

CO_2_ pneumoperitoneum with a stable abdominal pressure of 1.2 kPa and flow of 1.5 kPa, accompanied with 40 to 45° Trendelenburg positioning were performed in all patients in RARC group after anesthesia induction. When bladder isolation, ureters blocking, and lymph node dissection finished, patients were returned to the horizontal position and deflation.

### General anesthesia and monitoring

After a recipient was placed supine in the operating room, routine monitoring was performed using electrocardiography, pulse oximetry, non-invasive blood pressure, and end-tidal CO_2_ (etCO_2_). In accordance with our protocol, anesthesia was induced with propofol and sufentanil, with tracheal intubation facilitated with Rocuronium bromide. Anesthesia was maintained with sevoflurane in a 60% oxygen-air mixture accompanied by continuous infusions of remifentanil and cisatracurium. Then, the direct arterial pressure was monitored by femoral arterial catheterization. To monitor central venous pressure, a central venous catheter inserted into the internal jugular vein was connected to a sensor.

### Measurements of cerebral rSO2 and intraoperative variables

NIRS can be used to detect changes in oxygenated and deoxygenated hemoglobin associated with brain tissue hypoxia and estimate changes in cerebral blood volume and flow. For cerebral oximetry, sensors were placed on the patient’s forehead bilaterally. Cerebral rSO_2_ values were obtained from an INVOS 5100C Cerebral/Somatic Oximeter System, which generates 2 wavelengths of infrared light (730 and 805 nm) and penetrates into the skull and cerebral tissues. The cerebral oximetry sensor is consisted of a light-emitting diode and 2 detectors located at distances of 30 and 40 mm from the light-emitting diode to avoid light attenuation or extracranial contamination. Cerebral rSO_2_ values were automatically recorded every 5–6 s from the digital output port of the monitor to the personal computer.

Measurements of heart rate (HR), mean arterial pressure (MAP), central venous pressure (CVP), etCO_2_ and cerebral oxygen were simultaneously performed after entering the operating room (T0; baseline), anesthesia induction (T1), Trendelenburg positioning (T2), 1 h, 2 h, and 3 h after Trendelenburg positioning (T3, T4, T5 respectively), and after desufflation in a horizontal position (T6). We obtained arterial blood gas and vein blood gas for measurements of hemoglobin (Hb), serum glucose, sodium, potassium, base excess, lactic acid, PaCO_2_, PaO_2_, PvCO_2_, and PvO_2_ before the operation, and 10 min after desufflation.

### Neuropsychological assessment

We used Z score to analyze and comprehensively evaluate POCD [19]. Six cognitive scores (MMSE, BVMT-R, Symbol Digit Modalities Test, TMT, DST, and SCWT) [20] were combined into a composite cognitive score by averaging the Z scores of each test from the patients’ preoperative assessments. Thus, by definition, the mean preoperative score is 0 and the standard deviation is 1. When there were significant findings (≥ 1.96) in postoperative neurocognitive tests or the total Z score ≥ 1.96, then the patients were defined as POCD [15]. In this calculation, timed test scores were inverted to be consistent with non-timed tests, such that higher values represent superior performance for all tests.

### Statistical analysis

Data are presented as mean ± standard deviation (SD). With Comparing the incidence rate of POCD, the concentration of CRP and s100β between RARC group and control group was compared using the one-tailed Fisher’s exact test and independent sample t-test. Nonparametric test (Kruskal-Wallis test) was used for the cerebral oxygen saturation value and vital signs of RARC group during different time points. *P*-value of less than 0.05 was statistically considered significant. Statistical analysis was performed using the GraphPad Prism 5.01 software (GraphPad Inc., CA, USA).

## Result

### Demographic factors and intraoperative variables

A total of 50 patients were initially enrolled in this study, but 3 patients (1 in RARC group and 2 in the control group) were dropped out because of failure in data collection. Other 47 patients, including 24 cases in RARC group and 23 patients in the control group were enrolled as shown in Table 1. All operations and neuropsychological tests carried out well. Patients’ characteristics, including age, sex, body mass index (BMI), education status, Basal MMSE, Basal rSO_2_, and American Standards Association (ASA) classes II–III, were not different between the two groups (*P* > 0.05, Table 1). General data of surgery, including duration of Trendelenburg and operation, blood loss, and input fluid volume did not contain significant difference between those two groups (*P* > 0.05, Table 1). There were no significant differences in HR, CVP, rSO_2_, and SpO_2_ between two groups during surgery, while MAP was different at the point of Trendelenburg position and pneumoperitoneum (T1 vs. T2) (71.92 ± 7.751 vs. 93.25 ± 6.771, *P* < 0.05, Table 2). The etCO_2_ increased as the duration of Trendelenburg position and pneumoperitoneum prolonged (T2 vs. T5) (from 33.92 ± 1.881 to 44.00 ± 5.461, *P* < 0.05).

**Table 1:** Patients’ characteristics (mean ± SD)

Characteristic	RARC group (*n* = 24)	Control group (*n* = 23)	*P-value*
Sex, M/F	20/4	16/7	0.277
Age, y	67.75 ± 7.385	65.35 ± 4.097	0.177
BMI, kg/m^2^	23.26 ± 3.298	22.23 ± 2.430	0.232
ASA	2.000 ± 0.135	1.870 ± 0.114	0.466
Education status	2.667 ± 1.050	2.174 ± 0.937	0.097
Basal MMSE	28.75 ± 0.235	28.61 ± 0.279	0.700
Basal rSO_2_	63.93 ± 5.175	64.63 ± 3.363	0.697
Trendelenburg time/ Operation time, min	251.8 ± 69.72	227.4 ± 47.92	0.171
Blood loss, mL	166.0 ± 132.7	130.9 ± 110.1	0.326
Input fluid, mL	2204 ± 585.8	2043 ± 519.0	0.322

Education status: 1 = elementary school; 2 = middle school; 3 = high school; 4 = college/university. *P*-value is compared between the RARC group and control group

**Table 2:** rSO_2_/SpO_2_/etCO_2_/MAP/HR/CVP of the sample in RARC group (mean ± SD)

	rSO_2_ (%)	SpO_2_ (%)	P_et_CO_2_ (mmHg)	MAP (mmHg)	HR (bpm)	CVP (mmH2O)
T0	63.06 ± 3.222	99.22 ± 1.095		99.08 ± 13.03	70.42 ± 5.915	7.500 ± 0.798
T1	63.34 ± 4.008	99.39 ± 1.196	30.50 ± 1.087	71.92 ± 7.751	63.83 ± 11.53	8.167 ± 1.642
T2	65.80 ± 3.768	99.91 ± 0.288	33.92 ± 1.881	93.25 ± 6.771*^a^	59.67 ± 10.30	7.833 ± 1.801
T3	67.09 ± 4.345	99.91 ± 0.288	39.83 ± 3.786	78.67 ± 7.499	63.42 ± 4.358	9.333 ± 2.188
T4	67.99 ± 4.488	99.83 ± 0.388	41.67 ± 5.033	76.25 ± 5.818	67.83 ± 8.089	9.000 ± 1.954
T5	68.58 ± 3.510	99.78 ± 0.518	44.00 ± 5.461*^b^	78.56 ± 6.659	68.83 ± 7.493	9.333 ± 2.103
T6	67.61 ± 3.351	99.87 ± 0.458	41.42 ± 5.869	77.88 ± 7.159	72.50 ± 7.972	10.08 ± 1.621
T7	67.05 ± 2.937	100 ± 0.0	36.00 ± 2.954	83.88 ± 8.794	66.75 ± 6.538	10.33 ± 1.557

T0 = Baseline; T1 = anesthesia induction; T2 = Trendelenburg position and pneumoperitoneum; T3 = 1 h after Trendelenburg position and pneumoperitoneum; T4 = 2 h after Trendelenburg position and pneumoperitoneum; T5 = 3 h after Trendelenburg position and pneumoperitoneum; T6 = repositioning; T7 = end of procedure; *a = significance of difference between T1 and T2 (*P*<0.05); *b = significance of difference between T2 and T5 (*P*<0.05)

rSO_2_ = regional cerebral oxygen saturation measured with INVOS; SpO_2_ = peripheral oxygen saturation; P_et_CO_2_ = end-expiratory CO_2_ partial pressure; bpm = beats per min

We obtained arterial blood gas and vein blood gas before the operation and 10 min after desufflation. Our results showed that there was no significant difference between two groups preoperatively (Table 3). However, the differences in the lactic acid (1.421 ± 0.356 vs. 1.131 ± 0.296, *P* = 0.030), PaCO_2_ and PvCO_2_ (43.08 ± 4.057 vs. 39.37 ± 4.417, *P* = 0.032; 50.21 ± 5.047 vs. 46.12 ± 3.779, *P* = 0.028) were significantly increased 10 min after desufflation in RARC group in contrast to the control group. The other variables didn’t have any significant difference (Table 4).

**Table 3:** Preoperative and Postoperative arterial/vein blood gas

	Preoperative			Postoperative		
	RARC group (n = 24)	Control group (n = 23)	*P-value*	RARC group (*n* = 24)	Control group (*n* = 23)	*P-value*
pH	7.430 ± 0.037	7.416 ± 0.046	0.405	7.351 ± 0.051	7.326 ± 0.107	0.442
Hb	11.02 ± 1.879	11.26 ± 2.276	0.767	10.72 ± 1.205	11.13 ± 1.933	0.512
Glu	6.050 ± 0.836	7.469 ± 2.661	0.069	7.829 ± 1.312	9.462 ± 2.460	0.050
Lac	1.143 ± 0.388	0.977 ± 0.277	0.216	1.421 ± 0.356	1.131 ± 0.296	0.030^*^
Na^+^	139.5 ± 2.210	137.8 ± 3.313	0.137	142.7 ± 3.474	140.5 ± 2.961	0.093
K^+^	3.200 ± 0.353	3.469 ± 0.382	0.068	3.943 ± 0.462	3.938 ± 0.741	0.985
BE	0.571 ± 1.823	−0.554 ± 1.76	0.116	−2.021 ± 2.010	−3.415 ± 1.918	0.078
PaO_2_	237.6 ± 37.44	259.4 ± 34.55	0.129	238.1 ± 36.22	246.5 ± 49.10	0.618
PaCO_2_	37.54 ± 4.057	37.41 ± 5.862	0.948	43.08 ± 4.057	39.37 ± 4.417	0.032^*^
PvO_2_	54.31 ± 10.68	54.33 ± 0.688	0.994	56.23 ± 8.563	53.83 ± 6.124	0.414
PvCO_2_	44.82 ± 3.501	44.01 ± 3.994	0.589	50.21 ± 5.047	46.12 ± 3.779	0.028^*^

* = statistical significance of difference between RARC group and control group (*P*<0.05)

**Table 4:** Neuropsychological assessment

	RARC group (*n* = 24)	Control group (*n* = 23)	*P-value*
MMSE			
Postoperative	28.76 ± 1.128	28.77 ± 1.110	0.969
1 week after operation	28.00 ± 1.383	28.57 ± 1.076	0.133
3 months after operation	28.44 ± 0.768	28.55 ± 1.050	0.687
Digit Span Test			
Postoperative	8.500 ± 1.022	8.000 ± 1.214	0.145
1 week after operation	7.875 ± 1.296	7.238 ± 1.136	0.088
3 months after operation	8.000 ± 1.113	7.722 ± 0.958	0.409
Symbol Digit Modalities Test			
Postoperative	32.05 ± 7.599	30.25 ± 10.21	0.525
1 week after operation	27.70 ± 8.525	29.95 ± 12.23	0.483
Trail Making Test (sec)			
Postoperative	129.0 ± 41.83	109.3 ± 26.97	0.100
1 week after operation	153.5 ± 64.30	116.1 ± 60.39	0.045^*^
Brief Visuospatial Memory Test			
Postoperative	10.42 ± 2.099	11.45 ± 1.697	0.074
1 week after operation	10.69 ± 2.600	10.26 ± 1.982	0.566
Stroop Color and Word Test			
Postoperative	32.60 ± 12.29	33.00 ± 9.401	0.911
1 week after operation	29.44 ± 9.071	30.75 ± 11.07	0.766
Incidence of POCD	11/24	6/23	0.135

*MMSE* mini-mental state examination, *POCD* postoperative cognitive dysfunction. * = statistical significance of difference between RARC group and control group (*P*<0.05)

### Cerebral oxygen saturation

**Fig. 1:**
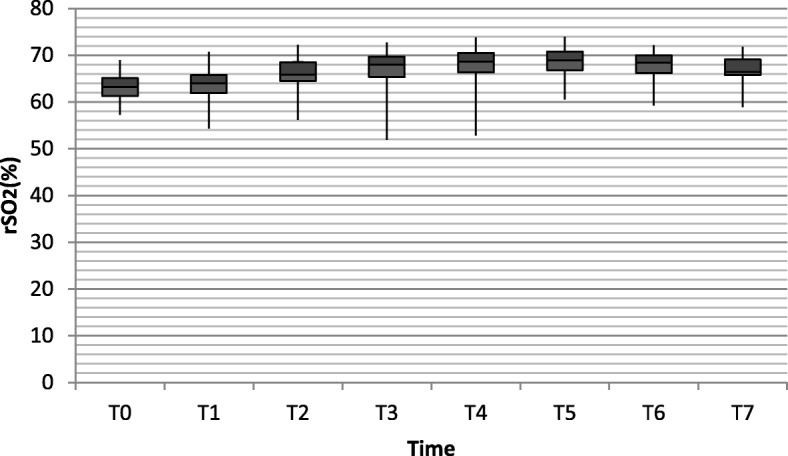
The cerebral oxygen saturation in RARC group at different time points. (T0 = Baseline; T1 = anesthesia induction; T2 = Trendelenburg position and pneumoperitoneum; T3 = 1 h after Trendelenburg position and pneumoperitoneum; T4 = 2 h after Trendelenburg position and pneumoperitoneum; T5 = 3 h after Trendelenburg position and pneumoperitoneum; T6 = repositioning; T7 = end of procedure)

**Fig. 2:**
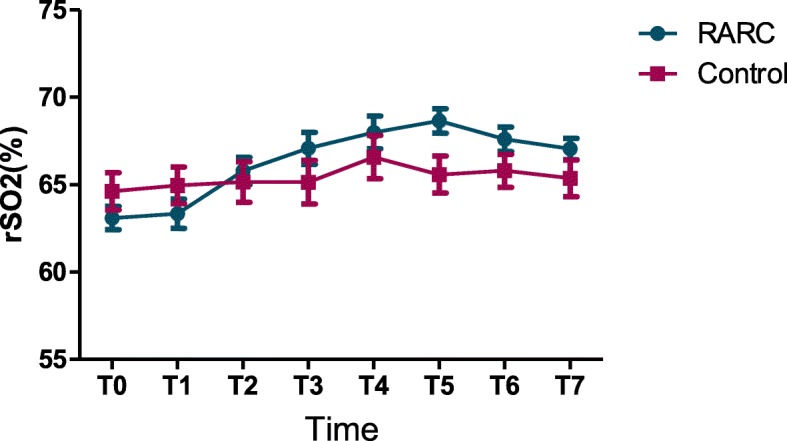
The cerebral oxygen saturation in RARC group and the control group. rSO2 values increased as duration of Trendelenburg position and pneumoperitoneum prolonged in the RARC group

The average basic rSO_2_ monitored by the INVOS 5100C cerebral oximeter system was 63.06 ± 3.222 in RARC group and 64.63 ± 3.363 in the control group (*P* = 0.697). In comparison with the control group, rSO_2_ value increased as duration of Trendelenburg position and pneumoperitoneum was prolonged in the RARC group (from 65.80 ± 3.768 to 68.58 ± 3.510). However, all the rSO_2_ values remained well below the threshold value of 75%, demonstrating that cerebral excessive perfusion is very unlikely. After repositioning, rSO_2_ gradually decreased (Table 2, Figs. 1 and 2).

### Neuropsychological tests

Herein, 11 patients were diagnosed as whom having POCD after 7 d in the RARC group, and the incidence rate of POCD was 45.83%. In the control group, 6 patients had POCD and the incidence rate of POCD was 26.09%. No significant difference in the incidence rate of POCD between the two groups (OR [95%CI], 0.42 [0.12–1.43]; *P* = 0.135) was achieved. However, the TMT’s score 1 week after operation in RARC group was significantly higher than the control group (153.5 ± 64.30 vs. 116.1 ± 60.39, *P* = 0.045, Table 4). We listed several probable risk factors of POCD, and we found that age (69.20 ± 7.033 vs. 65.34 ± 5.228, *P* = 0.041) and postoperative CRP (72.59 ± 42.09 vs. 48.50 ± 26.53, *P* = 0.025) had significant difference between patients with and without POCD postoperatively (Table 6).

### Blood assay

s100β significantly increased at stage of 48 h after operation compared with the preoperative stage in both groups (295.4 ± 172.9 vs. 110.2 ± 45.79, *P* < 0.001; 258.9 ± 179.1 vs. 99.80 ± 57.10, *P* < 0.001). Besides, CRP increased at stage of 48 h after operation in both groups (54.09 ± 38.80 vs. 2.083 ± 3.221, *P* < 0.001; 61.06 ± 29.33 vs. 2.448 ± 2.987, *P* < 0.001). Before surgery and 48 h after surgery, s100β and CRP had no significant difference between RARC group and the control group (*P* > 0.05, Table 5). There were no significant differences in the serum concentrations of s100β between patients with and without POCD postoperatively (*P* > 0.05, Table 6), while a significant difference was found in the serum concentrations of CRP (72.59 ± 42.09 vs. 48.50 ± 26.53, *P* = 0.025).

**Table 5:** CRP/s100β of the sample (mean ± SD)

	RARC group (n = 24)	Control group (n = 23)	*P-value*
CRP			
Preoperative	2.083 ± 3.221	2.448 ± 2.987	0.689
Postoperative	54.09 ± 38.80	61.06 ± 29.33	0.513
s100β			
Preoperative	110.2 ± 45.79	99.80 ± 57.10	0.499
Postoperative	295.4 ± 172.9	258.9 ± 179.1	0.486

**Table 6:** Risk factors of POCD

	POCD (*n* = 17)	non-POCD (*n* = 30)	*P-value*
CRP			
Preoperative	2.243 ± 2.691	2.271 ± 3.306	0.977
Postoperative	72.59 ± 42.09	48.50 ± 26.53	0.025^*^
s100β			
Preoperative	115.3 ± 73.29	99.48 ± 35.17	0.3248
Postoperative	327.0 ± 225.5	250.6 ± 138.3	0.161
Age	69.20 ± 7.033	65.34 ± 5.228	0.041^*^
Education status	2.412 ± 0.939	2.389 ± 1.086	0.938
Blood loss, ml	176.5 ± 163.8	132.6 ± 93.20	0.241
Input fluid, ml	2044 ± 505.9	2173 ± 582.9	0.449

Education status: 1 = elementary school; 2 = middle school; 3 = high school; 4 = college/university. * = statistical significance of difference between POCD group and non-POCD group (*P*<0.05)

## Discussion

### POCD

POCD was initially recognized as a common neurological complication secondary to anesthesia and surgery in 1955. The pathogenesis of POCD has not been properly clarified, and there are not sufficient validated risk models for POCD. Advanced age has been considered as an independent risk factor for developing POCD in several large cohort studies. The other risk factors mainly involve larger and more invasive operations, depth and duration of anesthesia, inflammation, change of ICP and CPP during surgery, etc. This is in agreement with the results of our study that age and postoperative CRP are independent risk factors of POCD.

Approximately 12% of patients who underwent anaesthesia and non-cardiac surgery will develop symptoms of cognitive dysfunction after their procedures [18]. In our study, the incidence rate of POCD in RARC reached up to 45.8%, which was higher than the laparotomy group. However, no significance was achieved even with a relatively substantial odds ratio. The wide range of the confidential interval may be explained by the limited case numbers included in our study. Considering the present findings, the association between POCD and RARC in this pilot study is not explicit. Nevertheless, to a certain extent, our results make sense to the necessity of further large sample of clinical studies.

In addition, the scores of MMSE and DST 3 months after surgery were both improved in RARC group and control group, which demonstrated that the symptoms of cognitive dysfunction postoperatively might occur in a relatively short period of time, and long-term postoperative cognitive dysfunction was not observed in the present study.

### NIRS

NIRS is used to assess regional cerebral oxygen saturation, presenting an indication for the balance between cerebral oxygen supply and consumption. Kalmar et al.’s study [11] reported that the rSO_2_ significantly increased when NIRS assessments were made with the subjects placed from the supine to the 20° Trendelenburg position. Similarly, in the present study, 40°~ 45° Trendelenburg position and CO_2_ pneumoperitoneum were associated with increase of rSO_2_ values in the brain. Throughout the duration of the procedure, rSO_2_ stayed well above the threshold value of 55% in each patient. At the beginning of Trendelenburg position and CO_2_ insufflation, rSO_2_ slightly increased. And as the time prolonged, the rSO_2_ value further increased. This was probably made by a combination of increased or unchanged CPP and an increase in PEtCO_2_, together resulting in vasodilatation and increase of cerebral blood flow as well as a decrease in oxygen extraction ratio. Among patients who participated in our study, rSO_2_ values were all below 75%, reflecting that Trendelenburg positioning and CO_2_ pneumoperitoneum didn’t cause excessive perfusion. The effect of positioning and CO_2_ insufflation was not strong enough to break the coordination of cerebral oxygen supply and demand.

### s100β and CRP

s100β and CRP have been shown to be increased in patients with cognitive dysfunction [21, 22] after cardiac surgery. Even so, S100β and CRP are not peculiar to brain tissue, which affect their diagnostic sensitivity [23, 24]. Studies about the relationship between plasma S100β or CRP and POCD after non-cardiac surgery indicated inconclusive results [25]. Our findings demonstrate that s100β and CRP were elevated 48 h after RARC and open abdominal surgery. In this study, it was revealed that there is no significant difference in s100β and CRP between RARC and control group. These results indicated that extreme Trendelenburg positioning and CO_2_ pneumoperitoneum might not lead to prominently severer inflammatory response and brain injury than those open abdominal surgery in supine positioning. That might also be related to small specimens and false-positive rate. We also found that there is not a strong relationship between S100βand cognitive dysfunction. Elevated CRP contributed to the occurrence of POCD in our study since postoperative CRP values increased significantly in the POCD group in Table 6.

### Limitation

There are several limitations in the present pilot study. The crucial constraints of this study include small sample size and nonparallel control. The study seems to suggest a higher incidence rate of POCD in RARC group compared to control group, but without significant difference, which might be attributable to small sample size. Moreover, small sample size may weaken the importance of the inflammation marker CRP in prediction of POCD. Although CRP in POCD group was higher 48 h after surgery than non-POCD group, its identity of reliable predictors of POCD still requires further research because we are not sure about the number of patients for statistical power and repeatability. In further study with enlarged sample size, the statistical analysis should be upgraded to a regression modeling to see whether any of the variables are predictive of POCD.

Considering small sample size and single-center study, the *P* value was set at 0.05. But the *P* value of education status was less than 0.1, showing a certain difference, which was probably a confounding factor in our study.

Besides, the follow-up visit of the incidence of POCD was a relatively short period of time. Lack of long-term follow-up visit may weaken the evidence of the information.

Last but not least, the Trendelenburg position might change the proportion between intracranial and extracranial blood detection inevitably. So the signal was probably inaccurate because of blood from extracranial arteries.

## Conclusion

Based on the existing sample size, no statistical significance was found to support the hypothesis that RARC increased the incidence of POCD. Extreme Trendelenburg positioning and CO_2_ pneumoperitoneum in RARC increased the cerebral oxygen saturation, whereas didn’t cause excessive perfusion. The inflammation marker CRP and age might be independent risk factors of POCD. More samples and further studies should be put forward to investigate the mechanism and other risk factors.

## Data Availability

The datasets generated and analyzed during the current study are available from the corresponding author on reasonable request.

## Abbreviations

ABG: Arterial blood gas
ASA: American Standards Association
BMI: Body mass index
BVMT-R: Brief Visuospatial Memory Test–Revised
CRP: C-reactive protein
CVP: Central venous pressure
DST: Digit Span Test
etCO_2_: End-tidal CO_2_
Hb: Hemoglobin
HR: Heart rate
IAP: Intra-abdominal pressure
ICP: Intracranial pressure
MAP: Mean arterial pressure
MMSE: Mini-mental state examination
MODS: Multiple organ dysfunction syndrome
NIRS: Near-infrared spectroscopy
POCD: Postoperative cognitive dysfunction
RARC: Robot-assisted radical cystectomy
rSO_2_: Regional cerebral oxygen saturation
SCWT: Stroop Color and Word Test
TMT: Trail Making Test
